# The relationship between the Geriatric Nutritional Risk Index and all-cause mortality in patients with peripheral artery disease

**DOI:** 10.1371/journal.pone.0325938

**Published:** 2025-06-27

**Authors:** Zhe Wu, Yue Yu, Bin Wang

**Affiliations:** 1 The First Clinical College, Shandong University of Traditional Chinese Medicine, Jinan, China; 2 The Traditional Chinese Medicine College, Shandong University of Traditional Chinese Medicine, Jinan, China; 3 Department of Vascular Surgery, The Second Affiliated Hospital of Shandong University of Traditional Chinese Medicine, Jinan, China; Necmettin Erbakan Üniversitesi: Necmettin Erbakan Universitesi, TÜRKIYE

## Abstract

**Background:**

Peripheral artery disease (PAD) is a common atherosclerotic condition that leads to limb dysfunction and increases mortality risk. Malnutrition is closely related to the long-term mortality of PAD patients. Therefore, studying the relationship between the Geriatric Nutritional Risk Index (GNRI) and long-term mortality in patients with PAD is crucial for identifying high-risk populations and developing targeted interventions.

**Methods:**

Data were sourced from the National Health and Nutrition Examination Survey (NHANES) conducted between 1999–2004, including 532 PAD patients. Kaplan-Meier survival analysis and multivariate Cox regression models assessed the relationship between GNRI and all-cause mortality in PAD patients. Subgroup analyses were conducted to explore differences based on demographic and disease backgrounds.

**Results:**

During the follow-up period, a total of 415 all-cause deaths were recorded. The Kaplan-Meier survival curve showed significant differences in mortality rates between the different GNRI quartile groups. Multivariate Cox regression analysis showed a significant negative correlation between GNRI and the long-term mortality risk of PAD patients (HR: 0.950, 95%CI: 0.918, 0.983). Compared to the first GNRI quartile, PAD patients in the third (HR: 0.569, 95%CI: 0.357, 0.909) and fourth (HR: 0.396, 95%CI: 0.208, 0.751) quartiles had a significantly reduced risk of long-term mortality. Restrictive cubic spline analysis showed a significant linear negative correlation between GNRI and all-cause mortality in PAD patients. The subgroup analysis results showed that the negative correlation between GNRI and all-cause mortality in PAD patients was significant in all subgroups except for the female subgroup, subgroup with ABI > 0.7, subgroup without smoking history, and subgroup without hypertension.

**Conclusion:**

There is a significant negative association between GNRI and all-cause mortality in PAD patients, suggesting that malnutrition may be a key factor affecting the prognosis of PAD patients. Early identification and intervention for malnutrition could reduce long-term mortality risks. Future research should further explore the role of nutritional interventions in the management of PAD and validate the findings of this study.

## Introduction

Peripheral artery disease (PAD) is a common atherosclerotic condition that predominantly affects middle-aged and elderly individuals [1–3]. PAD patients often experience symptoms such as coldness in the lower limbs, intermittent claudication, and foot necrosis, significantly impacting their mobility and quality of life [4–6]. According to research from the World Health Organization, the global population of elderly individuals is projected to exceed 2 billion by 2050 [7]. The ongoing progression of global population aging underscores the importance of addressing PAD as a major public health issue.

Nutritional status is increasingly recognized as a key factor influencing health outcomes in various chronic diseases [8–10]. Malnutrition is a prevalent issue among the elderly [11,12]. Research has shown that malnutrition can lead to adverse consequences such as decreased wound healing speed and increased susceptibility to infection in elderly people [13,14]. Consequently, PAD patients with poor nutritional status face a higher long-term mortality risk. The Geriatric Nutritional Risk Index (GNRI) is a tool used to assess nutritional risk in elderly patients [15]. It combines serum albumin levels and body weight measurements, providing a simple yet effective means of evaluating nutritional status. Lower GNRI scores indicate higher nutritional risk. However, the specific impact of GNRI on mortality among PAD patients in the general U.S. population remains underexplored.

Understanding the relationship between GNRI and mortality in PAD patients is crucial for identifying high-risk populations and developing targeted interventions. NHANES 1999–2004 provided relevant data for the study of peripheral arterial disease. In addition, NHAENS provides relevant follow-up data up to December 31, 2019, which allows for considerable follow-up time for research on PAD. Therefore, this study examines the association between GNRI and all-cause mortality in PAD patients using data from the National Health and Nutrition Examination Survey (NHANES) 1999–2004.

## Methods

### Data source

NHANES is an ongoing survey conducted by the National Center for Health Statistics (NCHS) to assess the nutritional and health status of the non-institutionalized U.S. population. The initial NHANES data was collected after ethical approval by the NCHS Institutional Review Board. All participants provided their written informed consent forms. Secondary analyses of publicly available data do not require further ethical approval.

**Fig 1:**
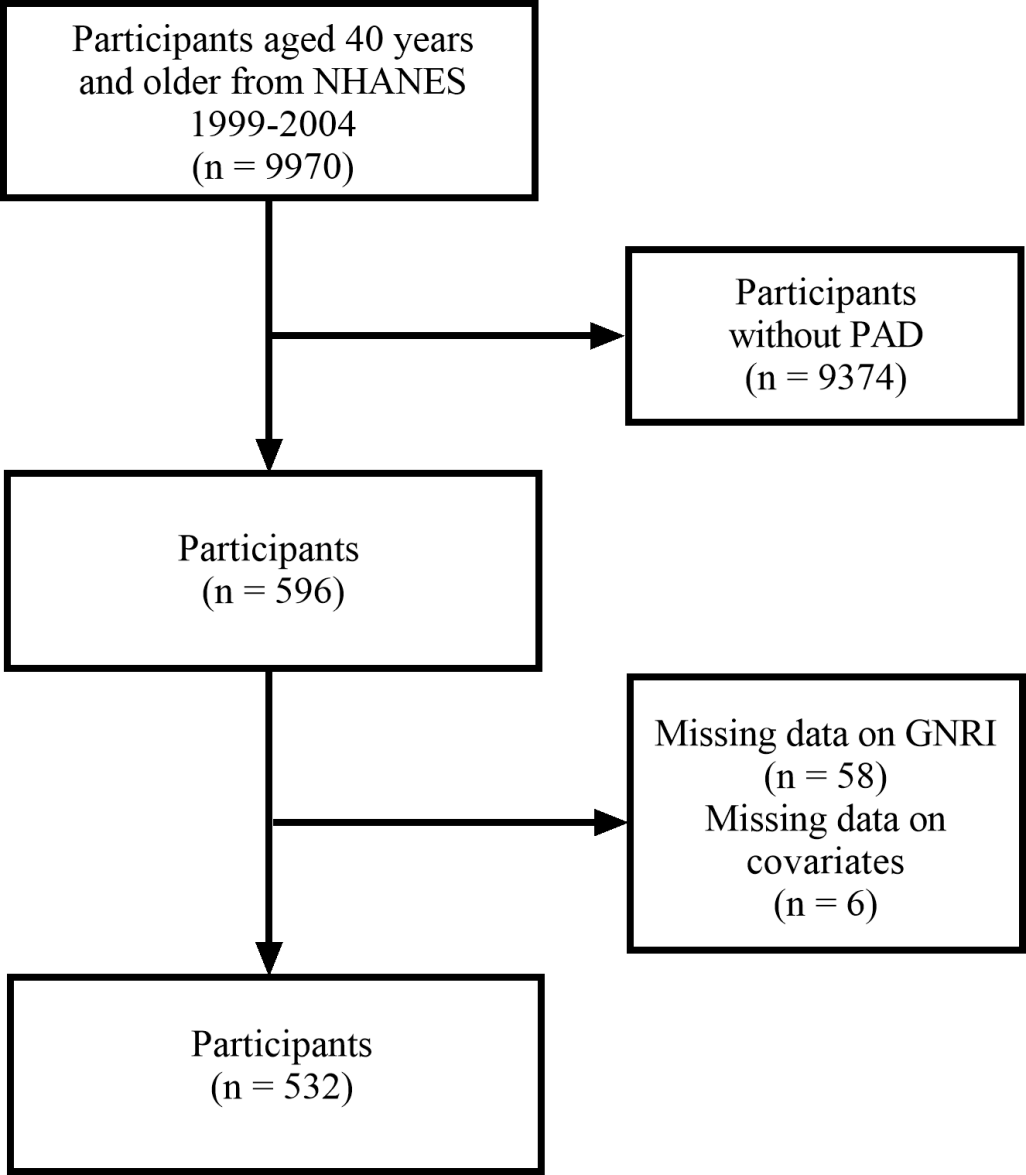
Data filtering process diagram.

In NHANES 1999–2004, 9970 participants aged 40 years and older underwent lower-extremity disease examinations. Among them, 596 were diagnosed with PAD. We excluded patients lacking GNRI data (n = 58) and those missing covariate data (n = 6), resulting in a final sample of 532 PAD patients (Fig 1).

### Peripheral artery disease

Systolic pressure was measured in the brachial artery of the right arm, or the left arm if the right arm could not be measured or if results could be affected. Systolic pressure was measured in both posterior tibial arteries at the ankles. The systolic ankle pressure for each side was divided by the systolic arm pressure to obtain the ankle-brachial index (ABI) for each side. PAD was diagnosed when ABI was less than 0.9 in at least one leg [16].

### GNRI

GNRI was calculated as follows:


$$GNRI=[1.489\times serum\;albumin\;(g/L)]+[41.7\times (actual\;weight\frac{kg}{ideal}weight\;(kg)]$$

Ideal weight was calculated using the formula H-100-[(H-150)/4] for males, and H-100-[(H-150)/2.5] for females. If a patient’s weight exceeded their ideal weight, the ratio of actual weight to ideal weight was set to 1 [15,17].

### All-cause mortality

All-cause mortality was determined using death records from the National Death Index (NDI). Survival status and follow-up data were collected up to December 31, 2019.

### Covariates

Covariates included age, sex, race, BMI, ABI, total cholesterol, aspartate aminotransferase (AST), alanine aminotransferase (ALT), smoking history, and the presence of hypertension, diabetes, cardiovascular disease (CVD), and chronic kidney disease (CKD). Hypertension was defined as an average systolic blood pressure of ≥140 mmHg, an average diastolic pressure of ≥90 mmHg, a doctor’s diagnosis, or the use of antihypertensive medications. Diabetes was defined by fasting glucose ≥7 mmol/L, random glucose ≥11.1 mmol/L, glycated hemoglobin ≥6.5%, a 2-hour OGTT glucose ≥11.1 mmol/L, or a doctor’s diagnosis or use of antidiabetic medications. CVD data were collected via questionnaires. CKD was defined as an estimated glomerular filtration rate <60 mL/min/1.73 m².

### Statistical analysis

Statistical analyses were conducted using R Studio (version 4.2.1), with all analyses weighted to account for population representation. Continuous variables are presented as means (standard error), while categorical variables are shown as means (weighted percentages). PAD patients were grouped into quartiles based on GNRI levels: Q1 ≤ 108.120, 108.120 < Q2 ≤ 113.847, 113.847 < Q3 ≤ 121.265, Q4 > 121.265. Kaplan-Meier (KM) survival analysis was used to examine long-term survival across different GNRI groups. A multivariate Cox regression model was employed to estimate the relationship between GNRI and all-cause mortality in PAD patients. Restricted cubic splines (RCS) were used to detect the dose-response relationship between GNRI and all-cause mortality in PAD patients. Subgroup analyses were conducted based on sex, race, smoking history, hypertension, diabetes, CVD, and CKD. Interaction effects were tested using the likelihood ratio test.

## Results

### Baseline characteristics

A total of 532 PAD patients were included in the study. The median follow-up time of the study was 118.5 months. During the follow-up period, a total of 415 all-cause deaths were recorded. Baseline characteristics of the included PAD patients, grouped by GNRI quartiles, are presented in Table 1. In addition, we grouped based on ABI levels (<0.5, 0.5–0.7, > 0.7). The baseline data grouped according to ABI level can be seen in S1 Table.

**Table 1:** Population characteristics stratified by GNRI.

Variable	Total	Q1	Q2	Q3	Q4
**Age (years)**	67.68(0.60)	71.25(1.33)	68.80(1.53)	69.10(1.35)	62.93(1.30)
**Sex**					
Male	268(44.12)	70(48.58)	75(44.11)	73(46.19)	50(39.13)
Female	264(55.88)	63(51.42)	58(55.89)	60(53.81)	83(60.87)
**Race**					
White	302(78.52)	76(77.21)	75(78.29)	80(80.71)	71(77.84)
Black	121(14.20)	26(12.55)	35(16.18)	23(10.35)	37(17.04)
Mexican Americans	86(3.36)	22(3.16)	19(2.94)	22(2.34)	23(4.71)
Other Race	23(3.92)	9(7.09)	4(2.60)	8(6.61)	2(0.41)
**BMI (kg/m**^**2**^)	28.50(0.34)	22.02(0.31)	25.60(0.32)	28.78(0.23)	35.41(0.67)
**ABI**	0.76(0.01)	0.73(0.02)	0.77(0.01)	0.74(0.01)	0.78(0.01)
**ALT (u/L)**	21.42(0.62)	21.05(1.74)	20.51(0.69)	21.11(1.58)	22.71(1.18)
**AST (u/L)**	23.70(0.59)	26.07(2.17)	22.93(0.64)	23.51(1.12)	22.76(0.78)
**Total cholesterol (mg/dL)**	210.28(2.49)	206.20(4.93)	217.20(6.94)	211.00(4.95)	206.96(4.39)
**Smoking history**					
No	175(32.55)	44(30.42)	42(32.22)	45(30.60)	44(36.03)
Yes	357(67.45)	89(69.58)	91(67.78)	88(69.40)	89(63.97)
**CKD**					
No	263(53.55)	60(47.39)	67(54.01)	61(49.91)	75(60.75)
Yes	269(46.45)	73(52.61)	66(45.99)	72(50.09)	58(39.25)
**Diabetes**					
No	371(73.81)	104(82.39)	93(70.68)	92(79.88)	82(64.97)
Yes	161(26.19)	29(17.61)	40(29.32)	41(20.12)	51(35.03)
**Hypertension**					
No	123(25.39)	34(27.14)	35(28.71)	27(25.18)	27(21.54)
Yes	409(74.61)	99(72.86)	98(71.29)	106(74.82)	106(78.46)
**CVD**					
No	349(64.55)	93(66.06)	84(61.90)	87(61.74)	85(68.00)
Yes	183(35.45)	40(33.94)	49(38.10)	46(38.26)	48(32.00)

### Kaplan-Meier analysis

**Fig 2:**
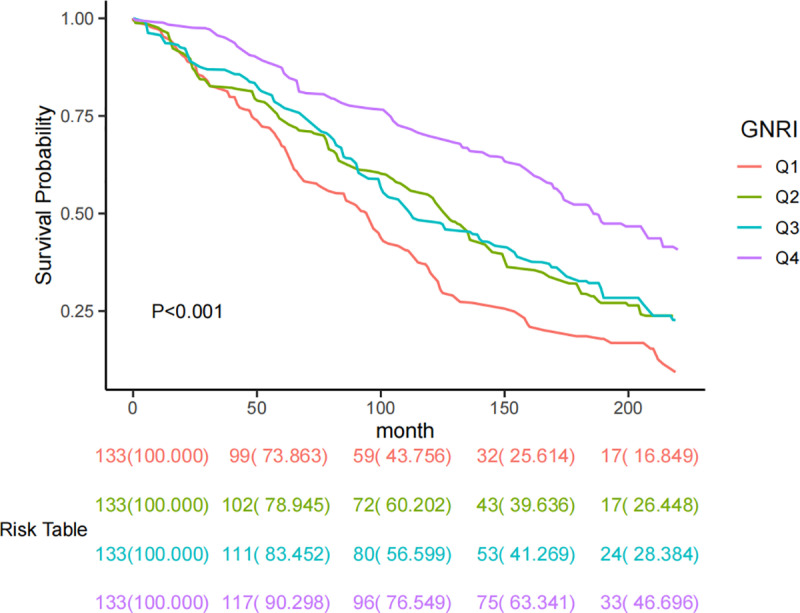
KM curve based on GNRI grouping.

The Kaplan-Meier curve demonstrated significant differences in mortality rates across the four GNRI quartile groups (p < 0.001) (Fig 2).

### Multivariate cox regression

Multivariate Cox regression analysis revealed a significant negative association between GNRI and all-cause mortality in PAD patients (HR: 0.950, 95%CI: 0.918, 0.983). Compared to the first GNRI quartile, PAD patients in the third (HR: 0.569, 95%CI: 0.357, 0.909) and fourth (HR: 0.396, 95%CI: 0.208, 0.751) quartiles had a significantly reduced risk of long-term mortality (Table 2).

**Table 2:** Relationship between GNRI and PAD.

Result	Model 1		Model 2		Model 3	
	HR (95%CI)	*P*-value	HR (95%CI)	*P*-value	HR (95%CI)	*P*-value
GNRI	0.972(0.960,0.983)	<0.001	0.983(0.970,0.996)	0.011	0.950(0.918,0.983)	0.003
Q1	Ref	Ref	Ref	Ref	Ref	Ref
Q2	0.689(0.481,0.989)	0.043	0.860(0.616,1.200)	0.374	0.810(0.609,1.077)	0.147
Q3	0.654(0.432,0.991)	0.045	0.720(0.488,1.062)	0.098	0.569(0.357,0.909)	0.018
Q4	0.386(0.262,0.568)	<0.001	0.598(0.394,0.909)	0.016	0.396(0.208,0.751)	0.005

Adjusted variables: Model 1: unadjusted. Model 2: age, sex, race. Model 3: age, sex, race, BMI, ABI, smoking history, total cholesterol, ALT, AST, diabetes, hypertension, CKD, CVD.

### Restricted cubic splines

**Fig 3:**
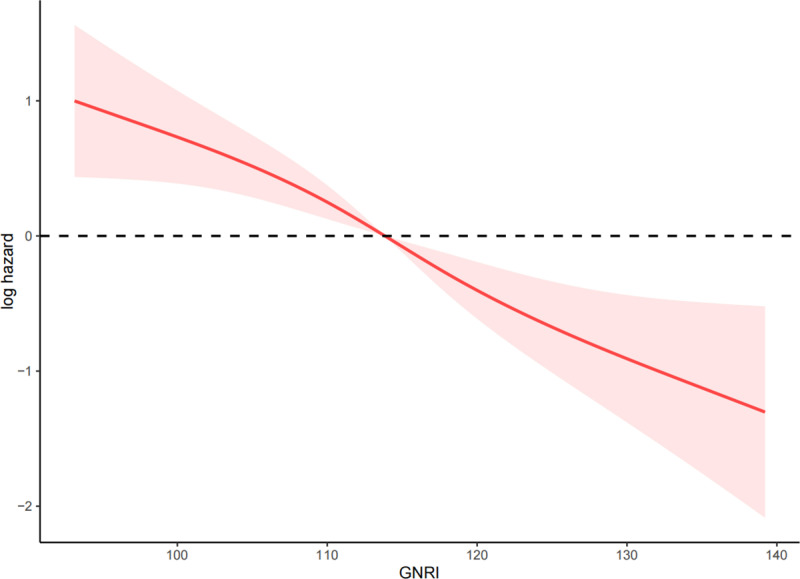
RCS of the relationship between GNRI and all-cause mortality in PAD patients.

The RCS analysis indicated a significant linear negative correlation between GNRI and all-cause mortality in PAD patients (P < 0.001, P for nonlinearity = 0.569) (Fig 3).

### Subgroup analysis

**Fig 4:**
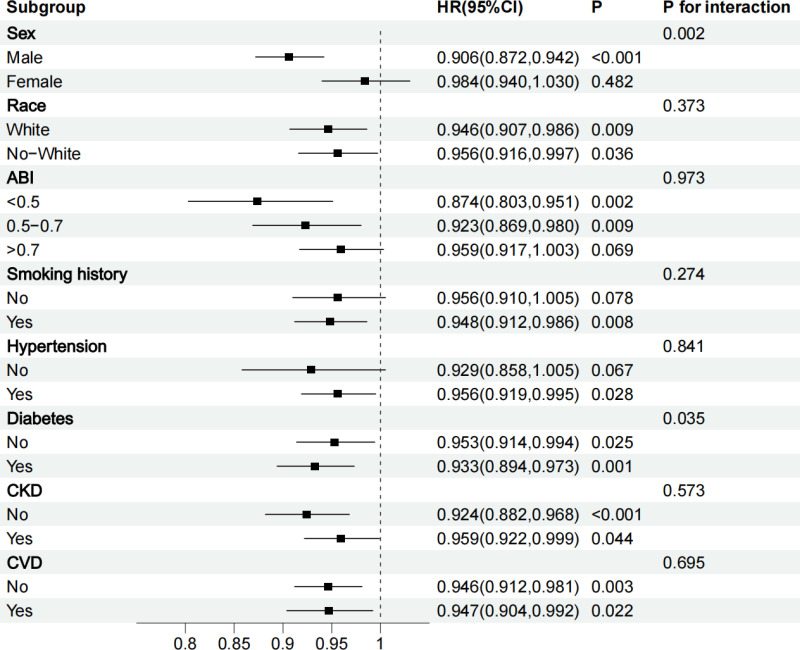
Forest map for subgroup analysis.

The subgroup analysis results showed that the negative correlation between GNRI and all-cause mortality in PAD patients was significant in all subgroups except for the female subgroup, subgroup with ABI > 0.7, subgroup without smoking history, and subgroup without hypertension. (Fig 4).

## Discussion

Our study is the first to explore the relationship between GNRI and all-cause mortality in PAD patients within the general U.S. population. The results show a significant negative association between GNRI and mortality risk in PAD patients. This suggests that poor nutritional status may be a key risk factor for adverse outcomes in PAD patients.

Our findings align with previous studies. A meta-analysis shows that the GNRI is an independent predictor of long-term mortality in patients with PAD [18]. Both using GNRI as a categorical variable and a continuous variable have shown a significant correlation between lower GNRI and increased all-cause mortality in PAD patients [19–24]. However, the literature included in this meta-analysis mainly focuses on patients with chronic limb-threatening ischemia (CLTI) who underwent revascularization surgery, and most of these studies are from Japan. Only one study, which focused on PAD patients with ABI < 0.9 rather than CLTI patients who merely underwent revascularization, found that low GNRI is an independent risk factor affecting the long – term survival of PAD patients [20].

Our study stands out as the first to examine the relationship between GNRI and all-cause mortality in PAD patients within the U.S. general population. Unlike previous research focusing on hospitalized patients, we selected participants from the general population and had a median follow-up of 118.5 months. The longest follow-up period of the study reached 247 months, ensuring the scientific and complete nature of the results. In addition, previous studies typically used a GNRI threshold of < 98 to define malnutrition. But in our study, we found that only about 6% of participants had a GNRI < 98. Therefore, we divided the GNRI scores into quartiles to better illustrate the effect of different GNRI levels on long-term mortality in PAD patients. This also demonstrates the importance of studying the relationship between GNRI and long-term mortality in PAD patients in different populations.

The increased long-term mortality risk in PAD patients with malnutrition may be attributed to several mechanisms. First, malnutrition weakens the body’s immune defense, with low serum albumin levels impairing the production and function of immune cells, particularly T-lymphocytes [25–27]. This immune suppression increases infection risk and may also trigger chronic low-grade inflammation. Moreover, malnutrition itself may lead to an increase in inflammatory response [28]. Inflammatory reaction is an important factor of atherosclerosis. Metabolic disturbances are another key mechanism linking malnutrition to elevated PAD mortality risk. Malnutrition can lead to insulin resistance and abnormal lipid metabolism, which are closely related to the development of atherosclerosis [29–32].

Nutritional interventions may be a key strategy for improving outcomes in PAD patients with low GNRI [33]. Research shows that appropriate nutritional support plays an important role in improving the prognosis of PAD patients [34]. Moreover, PAD patients often present with multiple comorbidities, such as hypertension, diabetes, and CKD, which are also associated with malnutrition [35–37]. Therefore, managing PAD patients should adopt a comprehensive approach, incorporating nutrition assessments and interventions alongside pharmacological treatments. In addition, GNRI is calculated solely based on albumin levels and body weight. Regular monitoring of GNRI levels in PAD patients can help detect malnutrition early and intervene promptly to reduce the risk of long-term mortality.

Our research also has limitations. First, as an observational study, it cannot establish causality. Second, although we adjusted for several confounders, unmeasured factors may still influence the results. In addition, PAD relies solely on ABI < 0.9 for diagnosis without relevant information on the severity of clinical symptoms, which limits the clinical interpretability of the results. Finally, we are unable to obtain information on whether PAD patients have undergone revascularization surgery and other related information, which may have an impact on the results.

## Conclusion

In summary, this study identified a significant negative association between GNRI and all-cause mortality in PAD patients, suggesting that malnutrition may be a critical factor influencing prognosis. Clinically, it is essential to assess and manage the nutritional status of PAD patients. Future research should further explore the role of nutritional interventions in PAD management and validate the findings of this study.

## Supporting information

S1 Raw data Original data for this study. (XLSX)

S1 Table Population characteristics stratified by ABI. (DOCX)
