# Supplementary material for: The relationship between the Geriatric Nutritional Risk Index and all-cause mortality in patients with peripheral artery disease
Source: PLoS One. 2025 Jun 27;20(6):e0325938. doi: 10.1371/journal.pone.0325938 (PMC12204543; doi:10.1371/journal.pone.0325938)
Supplement: S1 Table — (DOCX) [file pone.0325938.s002.docx]

| **S1 Table. Population characteristics stratified by ABI.** | | | | |
| --- | --- | --- | --- | --- |
| **Variable** | **Total**  **(n=532)** | **ABI < 0.5 (n=39)** | **0.5 ≤ ABI ≤ 0.7 (n=143)** | **ABI > 0.7**  **(n=350)** |
| **Age (years)** | 67.68(0.60) | 72.94(1.40) | 70.93(1.44) | 66.24(0.63) |
| **Sex** |  |  |  |  |
| Male | 268(44.12) | 18(49.36) | 84(54.89) | 166(40.33) |
| Female | 264(55.88) | 21(50.64) | 59(45.11) | 184(59.67) |
| **Race** |  |  |  |  |
| White | 302(78.52) | 20(78.26) | 75(71.85) | 207(80.64) |
| Black | 121(14.20) | 9(13.82) | 40(18.87) | 72(12.77) |
| Mexican Americans | 86( 3.36) | 9(4.07) | 21(4.21) | 56(3.03) |
| Other Race | 23( 3.92) | 1(3.85) | 7(5.06) | 15(3.56) |
| **BMI (kg/m^2^)** | 28.50(0.34) | 29.53(1.15) | 26.78(0.50) | 28.95(0.46) |
| **GNRI** | 116.08(0.64) | 115.08(2.16) | 112.97(1.02) | 117.13(0.86) |
| **ALT (u/L)** | 21.42(0.62) | 17.78(1.05) | 21.15(1.28) | 21.79(0.81) |
| **AST (u/L)** | 23.70(0.59) | 20.68(1.11) | 23.48(0.72) | 24.01(0.80) |
| **Total cholesterol (mg/dL)** | 210.28(2.49) | 190.82(8.52) | 215.33(3.87) | 210.23(2.93) |
| **Smoking history** |  |  |  |  |
| No | 175(32.55) | 10(26.71) | 39(26.08) | 126(35.04) |
| Yes | 357(67.45) | 29(73.29) | 104(73.92) | 224(64.96) |
| **CKD** |  |  |  |  |
| No | 263(53.55) | 13(25.62) | 61(47.28) | 189(57.71) |
| Yes | 269(46.45) | 26(74.38) | 82(52.72) | 161(42.29) |
| **Diabetes** |  |  |  |  |
| No | 371(73.81) | 24(62.57) | 95(73.55) | 252(74.77) |
| Yes | 161(26.19) | 15(37.43) | 48(26.45) | 98(25.23) |
| **Hypertension** |  |  |  |  |
| No | 123(25.39) | 3( 9.81) | 33(22.75) | 87(27.44) |
| Yes | 409(74.61) | 36(90.19) | 110(77.25) | 263(72.56) |
| **CVD** |  |  |  |  |
| No | 349(64.55) | 25(61.02) | 85(53.13) | 239(68.42) |
| Yes | 183(35.45) | 14(38.98) | 58(46.87) | 111(31.58) |
